# A Rare Kimura’s Disease in the Oral Cavity with Severe Sleep Apnea: Case Report and Literature Review

**DOI:** 10.3390/medicina58121810

**Published:** 2022-12-09

**Authors:** Xiaoyue Lei, Dan Yu, Xiaoyan Feng, Yiyang Shen, Huiyong Zhu

**Affiliations:** 1 Department of Oral and Maxillofacial Surgery, The First Affiliated Hospital of Zhejiang University School of Medicine, Hangzhou 310003, China; 2 Zhejiang University School of Medicine, Hangzhou 310058, China

**Keywords:** Kimura’s disease, oral cavity, radiotherapy, diagnosis

## Abstract

Kimura’s disease (KD) is a rare chronic inflammatory disorder that commonly occurs in Asian males. It mainly presents as painless subcutaneous masses or lymphadenopathy in the head and neck region. The incidence of KD in the oral cavity is quite rare. We reported a rare case of a 53-year-old male who had KD in his soft palate, hard palate and bilateral tonsils associated with severe sleep apnea. This patient underwent radiotherapy and exhibited a good response to the treatment. Throughout the 12-month follow-up period, the patient’s condition remained satisfactory. Of the other 14 reviewed cases of KD in the oral cavity, the lesions can occur in the buccal mucosa, hard and soft palate, and mouth floor with specific clinical features. We further summarized their manifestations and treatments in order to guide the future identification and management of KD with lesions in the oral cavity.

## 1. Introduction

Kimura’s disease (KD) is a rare, benign, chronic inflammatory disease of unknown etiology that typically presents with slowly enlarging, non-tender, subcutaneous masses. It is more prevalent in young Asian males, with a peak incidence in the second and third decades of life [1,2]. The disease was first reported by Kim in China as “eosinophilic hyperplastic lymphogranuloma” in 1937 [3] and has been widely known as KD after a detailed description by Kimura in 1948 [4]. KD is also characterized by peripheral eosinophilia and markedly elevated serum IgE. Lymphoid follicular hyperplasia, eosinophilic infiltration, vascular hyperplasia and lesion-surrounding fibrosis are pathological features of KD. As seen in a previous report, 73.9% of all KD is presented in the head and neck regions, predominantly in the salivary glands and regional lymph nodes [5]. The incidence of KD in the oral cavity is quite rare, and there are only a few reported cases of KD in the submucous tissue of the oral cavity. Here, we report a rare case of a mass that is extensively involved with the soft palate, the hard palate and the bilateral tonsils, and we discuss the current studies of KD with lesions in the oral cavity.

## 2. Detailed Case Description

A 53-year-old male presented with a 2-year history of painless swelling in the oral cavity and submandibular regions accompanied by severe sleep apnea. An excisional biopsy of the neck lymph node was taken at the previous hospital before the patient’s referral to our hospital, and the histology examination confirmed the diagnosis of KD. Oral prednisone therapy was chosen as the initial treatment for the patient in a previous hospital. After several months of steroid therapy, this patient found that the mass was reduced and that symptoms of sleeping apnea were alleviated. Then he stopped the drug without any guidance for fear of medical complications. However, a few months later, the patient again presented with the same symptoms as the lesion in the oral cavity progressed. His medical history revealed untreated mild hypertension and bilateral submandibular lymphadenectomy 20 years ago with unknown pathology. No renal dysfunction or other comorbidity was observed.

**Figure 1:**
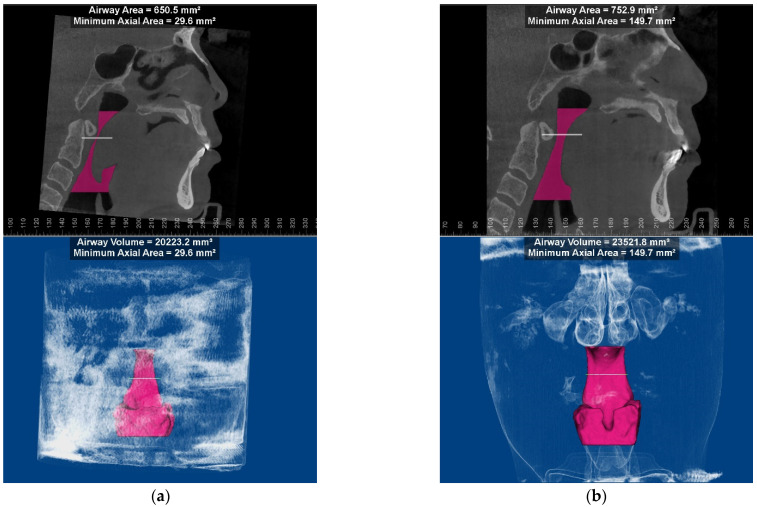
3D reconstruction and airway analysis conducted by Dolphin imaging software before RT (**a**) and at two months after radiation therapy (**b**).

Physical examination demonstrated a poorly defined and un-tender soft mass involving the hard palate, bilateral tonsils and nearly the entire soft palate. The mucosa was intact, and the lesion appeared a color of dark red with patchy brown pigmentation on the surface. A soft, painless swelling was observed in his bilateral submandibular area. Magnetic resonance imaging (MRI) revealed a mass with a size of 4.3 × 5.7 cm in his soft palate and bilateral tonsils, exhibiting a hypo-intense signal on T1-weighted images and a hyper-intense signal on T2-weighted images; multiple lymph nodes enlarged in the cervical and submandibular region and malignancy could not be ruled out by image findings. Cone beam computerized tomography (CBCT) demonstrated soft tissue thickening involving the undersurface of the posterior half of the hard palate, soft palate and bilateral tonsils with a slight cortical erosion in the hard palate. Blood tests showed an increased eosinophilia count of 3.15 × 10^9^/L (43.7% of the total white blood cells; reference range, 0.5–5%) and elevated serum IgE of >5000 IU/mL (reference range, 0–100 IU/mL). Other laboratory findings were within normal limits. The biopsy results of the soft palatal mass and neck lymph nodes (from the previous hospital) were both consistent with pathological features of KD, including reactive lymphoid follicles with eosinophilic micro-abscesses, numerous eosinophilic and lymphocytic infiltration, an increased amount of postcapillary venules, and varying degrees of fibrosis. Based on the clinical presentation, radiographic findings, laboratory examination and histopathological results, a definitive diagnosis of KD was made. Considering the difficulty in breathing reported by the patient, the CBCT data were further imported into Dolphin imaging 11.8 software in the DICOM format to quantify his upper airway volume, where the results showed that the minimum axial airway area was only 29.6 mm^2^ (Figure 1A). Additionally, the apnea–hypopnea index was 54.0/h by polysomnography, indicating he had severe obstructive sleep apnea (OSA).

**Figure 2:**
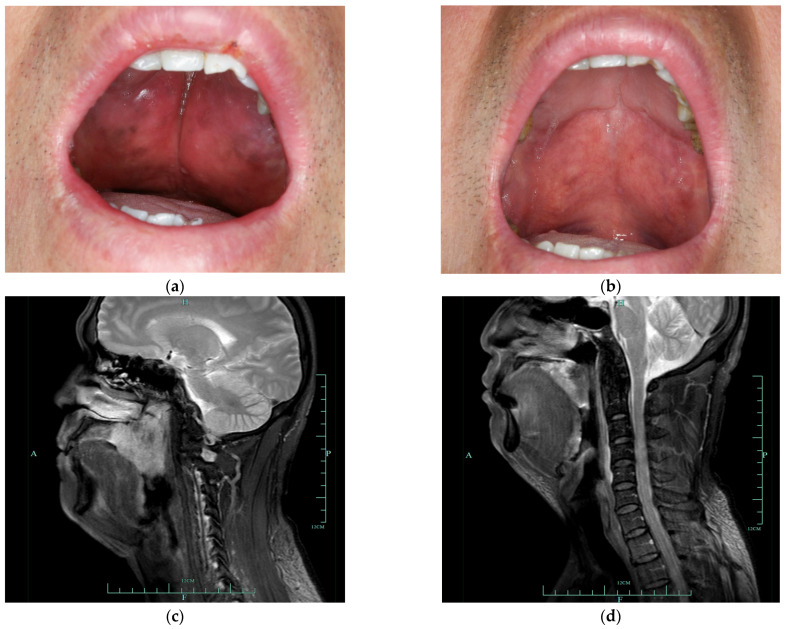
Intraoral photographs and MRI images of the mass before treatment (**a**,**c**) and at two months after radiotherapy (**b**,**d**).

Due to the wide infiltration of the lesion, total excision was not feasible, and we decided to perform a conservative treatment. This patient then underwent radiotherapy at a total dose of 3960 cGy, with 22 single doses of 180 cGy. Significant regression of the mass was observed both by clinical examination and MRI after radiotherapy (Figure 2), accompanied by the alleviation of sleep apnea. The minimum axial airway area was remarkably increased (149.7 mm^2^ vs. 29.6 mm^2^, Figure 1B) two months after radiotherapy. The patient was followed closely in the past 12 months, and no evidence of the lesion progressing was detected to date.

## 3. Discussion and Conclusions

Kimura disease is a benign, chronic inflammatory disease characterized by painless subcutaneous masses in head and neck regions with associated lymphadenopathy, increased blood peripheral eosinophilia and marked evaluated IgE [6]. The exact etiology mechanism of KD remains unclear, although inflammation, allergies, endocrine disorders, fungal and viral infections and autoimmune diseases have been reported to be the possible causes [7,8]. In this study, we reported a rare case of KD involving the hard palate, bilateral tonsils and nearly the entire soft palate and summarized the specific clinical findings from reported KD involving the oral cavity, which can help in the early identification of the condition.

Kimura’s disease commonly presents with a subcutaneous mass in the head and neck region, particularly involving the salivary gland (mainly parotid and submandibular glands) and regional lymph nodes. To the best of our knowledge, only 17 cases reported KD with lesions involving submucous tissue of the oral cavity. The basic information and treatments of these patients (including our patient) are described in Table 1. The male-to-female ratio of 18 cases was 3.5:1, similar to the reported sex distribution in all KD patients (male: female 3.5–6:1) [2,9]. The medium age at onset was 47, ranging from 3 to 63, older than the onset age reported by the previous literature [5,10]. Palate (61.1) was the most common location, followed by buccal mucosa. To be specific, six cases (33.3%) involved the hard palate, three cases (16.7%) involved the soft palate, two cases (11.1%) involved both the hard and soft palate, five cases (27.8%) involved the buccal region, one case (5.6%) involved the mouth floor and one case (5.6%) involved tongue. The duration of the lesions can range from 6 weeks to 10 years.

Because of its rarity in the oral cavity, both clinicians and radiologists are not familiar with oral manifestations of KD, which may lead to unnecessary diagnostic tests and investigations. Misdiagnosis was common, where four reported cases from the articles have considered the lesions as malignant tumors following the patients’ first clinical visit. Therefore, it is necessary to understand oral manifestations of KD to help with the early identification of the disease. The clinical courses of KD varied significantly among cases, but some specific clinical features were noted (Table 2). Most cases presented with an intact, painless, and slow-growing mass involving soft tissue. KD is often associated with pruritus or melanin pigmentation, probably due to nerve infiltration by lymphocytes and eosinophils [26]. Among the cases involving the oral cavity, distinctive mucosa pigmentation was observed in five cases (27.8%), while mucosa pruritus was recorded in one case (5.6%), and all of them were in hard or/and soft palate. One of them was even initially misdiagnosed as melanoma for the extensive poorly defined hypermelanotic mass [14]. It is worth mentioning that bone resorption in varying degrees was reported in three cases that involved the hard palate. One patient presented with an ulcerated mass with extensive bone destruction involving the alveolar ridge, hard palate and floor of the paranasal sinus for unknown reasons [19]. The other two patients [22] (including ours) reported subtle bone erosion which was possibly due to the mass compression. On experiment examinations, marked evaluated serum IgE levels have been reported in seven cases (38.9%), while two cases claimed normal IgE levels. Moreover, 11 patients (61.1%) had moderate to marked peripheral blood eosinophilia. KD can be a systemic and multi-organ disease. Four patients (22.2%) had subcutaneous masses outside the head and neck regions, predominantly in the axilla, groin and limbs, and three patients (16.7%) had a history of nephrotic syndrome. As renal disorder is the most common systemic manifestation of KD, with incidence rates ranging from 10% to 16% based on previous studies, the kidney routine examination was recommended for all KD patients [27]. Imaging manifestations of KD in the oral cavity were polymorphous and non-specific in those cases, although previous studies have demonstrated that the lesions commonly exhibit moderate to high signal intensity on T1-weighted images and high signal intensities on T2-weighted images [28]. In contrast-enhanced CT or MRI, the lesion usually had a moderate to high retention of contrast agents.

The characteristic symptoms described above can indicate a possible diagnosis of KD. However, diagnostic operations or biopsies are indispensable since the accurate diagnosis of KD requires pathological confirmation. The histological results of KD are characterized by lymphoid follicular hyperplasia with germinal center enlargement, eosinophilic infiltration, the accumulation of eosinophilic micro-abscesses, and postcapillary and venular hyperplasia, surrounded by circular collagenous fibrous deposition and differing extents of fibrosis [7]. Individually, those features are non-specific and can easily be found in other benign and malignant disorders that present lymphocytes with a prominence of eosinophils, including parasitic lymphadenopathy, angiolymphoid hyperplasia with eosinophilia, eosinophilic granuloma, Hodgkin lymphoma (HL), angioimmunoblastic T-cell lymphoma and Churg–Strauss syndrome. The differential diagnosis between those diseases requires a combination of clinical and pathological features as well as laboratory examination [29]. Moreover, the necessary immunohistochemical markers can be advantageous for assisting differential diagnosis, such as CD15 and CD30 in the atypical cells for the diagnosis of HL. In terms of prognosis, high expressions of the Notch-1 receptor and Ki-67 have been shown as potential predictors for KD recurrence [30].

The optimal treatment for KD has not yet been established. Several therapeutic options are suggested for KD, such as surgical excision, radiotherapy and medical therapy (including steroids and/or immunosuppressants) [31]. Surgery is widely used for diagnostic and therapeutic purposes. In patients with maximal tumor diameter <3 cm, surgery was regarded as an effective single-treatment modality. However, for patients with larger and infiltrative lesions, complete surgical removal is limited, and the unclear boundaries may lead to rapid recurrence [32]. Additionally, high-volume resections may contribute to cosmetic defects and functional disability. Corticosteroid therapy has been commonly used as a therapeutic strategy, especially for patients with multi-organ involvement, but recurrence frequently occurs while tapering the drug dose or withdrawing the medication [33]. Another concern regarding steroids is that their long-term use of them may lead to complications such as osteoporosis, digestive ulcers and acquired diabetes mellitus. Other systemic immunosuppressive medications, including cyclosporine [34], mycophenolate mofetil, mycophenolic acid [35], leflunomide and tacrolimus [36] were also reported to be effective in some patients. In a recent meta-analysis including 31 studies, the overall recurrence rate of surgical excision was 30.5%, and that of medication alone was 45%, while the combination therapy of surgery and postoperative adjuvant therapy demonstrated the lowest recurrence rate of 26.94% [32]. Therefore, postoperative adjuvant treatments (including radiotherapy and systemic immunosuppressive medications) are recommended for patients with the risk factors of recurrence, especially for those with positive margins. RT played a role as an exclusive treatment in the past, but it was more often employed in an adjuvant setting recently. Based on the results in earlier studies, the local control rate was between 74.1–100% for patients who received RT as a primary treatment or a secondary treatment for recurrence. The tolerance and side effects of RT have raised some concerns, but they can be avoided by advocating a low dose of radiation. There has been no report on radiation-induced carcinogenesis in KD [37]. Altogether, surgery is the primary treatment for resectable lesions in current practice, and combination adjuvant therapy with surgical excision is recommended for those with a high risk of recurrence. For patients with multi-organ involvement, a systemic glucocorticoid is a viable option [38].

In our case, the lesion invaded so widely that complete excision was not feasible. The patient has gone through recurrence after oral steroid therapy at a previous hospital, and he declined to take steroids for his condition. Finally, considering the lack of multiple site involvement and systematic symptoms, we decided to treat the patient with medium-dose RT and follow him up closely completion of treatment. Although the optimal RT dose remained inconsistent and the association between total dose and local control was not found yet, 20 Gy and 45 Gy with standard fractionation were commonly used for KD in head and neck regions without oral cavity involvement [39]. Radiation treatment for this patient was administered at a total dose of 3960 cGy, and the mass responded favorably to the treatment. During RT, treatments were tolerated extremely well by the patient, and no significant side effects were experienced. At the 12-month follow-up evaluation, there was no evidence of the lesion progressing. Previous reports of KD in the oral cavity also reported several effective treatments except for RT (Table 3). Six patients were given surgical therapy, and two of them received only partial excision due to the broad tumor extent. Moreover, five patients were given only medical therapy, and five patients received combination therapy of surgery and steroid. Among the two relapsing patients (11.1%), one had recurrence three months after limited surgery, and the other had recurrence three months after pentoxifylline withdrawal. A previous study has mentioned that the incidence of recurrence was in a mean follow-up time between 1.44 and 10.4 years, suggesting that the low recurrence rate from this review may be due to the short follow-up time [32]. Therefore, longer follow-up is needed to determine the long-term safety and efficacy of the treatment modalities for KD patients with oral cavity involvement.

Obstructive sleep apnea is a sleep-related breathing disorder that is often associated with a compromised upper airway space. Palatal, tongue or retropharyngeal soft tissues bulky or retroposition can cause OSA. Two patients (including ours) with diffusing masses in the soft palate had severe OSA. In addition to the oral cavity, OSA is more frequently presented in patients with KD involving epiglottis or vocal cords [40,41], where the lesion is relatively occult. Therefore, KD and other space-occupying lesions in the oral cavity and larynx should be excluded in patients with OSA [42]. Polysomnography remains the gold standard for diagnosing OSAS. However, it is very time-consuming, expensive and has spatial limitations. As an accessible and economical inspection, CBCT was employed in our patient to assess the minimum axial airway area and monitor changes in his upper airway. Although there is no unified quantitative classification of upper airway anatomical traits for the diagnosis of OSA, the minimum cross-sectional area of the upper airway has been widely considered to be associated with OSA [43]. Thus, three-dimensional reconstruction of CBCT is recommended to measure and monitor the airway for KD patients with OSA.

In conclusion, KD can occur in the oral cavity, including the hard and soft palate, buccal, tongue and mouth floor. It is necessary for maxillofacial surgeons to understand the clinical features of oral KD to reduce misdiagnosis and help with the early identification of the disease. Recognizing the histopathological features of KD and applying the appropriate biopsy is crucial in establishing the correct diagnosis. Although combination adjuvant therapy with surgical excision is preferred in current clinical practice, RT is still an effective therapy for patients with extensive local lesions to prevent unacceptable facial deformity. However, close and long-term follow-up is recommended due to the significant recurrence rate of KD.

## Figures and Tables

**Table 1:** Basic characteristics and imaging features of Kimura disease presented with masses in oral cavity.

Study	Age	Sex	Duration	Location	MRI	CT	Contrast Enhanced MRI/CT	Follow Up
Sato et al. [5]	52	Male	2 years	Buccal	T1: hypo-intense signal T2: iso-intense signal	NR	NR	NR
Lee et al. [11]	16	Male	3 years	Buccal	NR	Nodular enhancing lesion with perilesional soft-tissue infiltration	NR	12 months
Ahmed et al. [12]	3	Male	3 years	Buccal	NR	Nodular enhancing lesion with perilesional soft-tissue infiltration	NR	12 months
Hongcharu et al. [10]	36	Male	Several months	Buccal	NR	NR	NR	14 months
Bayır et al. [13]	57	Male	2 years	Buccal	T1: heterogeneously intense signal T2: hyperintense signal	NR	Higher utilization of contrast agent	12 months
Benslama et al. [14]	61	Male	Recently	Hard palate	No obvious abnormalities	No obvious abnormalities	NR	NR
Terakado et al. [15]	33	Male	10 years	Hard palate	T1: slight reduction of signals T2: slight reduction of signals	NR	NR	24 months
Ray et al. [16]	46	Male	2 years	Hard palate	NR	NR	NR	NR
Zhang R et al. [17]	53	Male	1 month	Hard palate	NR	Ill-defined mass with hypo density	Ill-defined mass in the left palate with a homogeneous moderate enhancement	>12 months
Dermatol C et al. [18]	40	Female	1 year for oral lesion	Hard palate	NR	Enhancing soft tissue masses and skin thickening	NR	Several months
Urs D. A. et al. [19]	56	Male	6 weeks	Hard palate and upper jaw	Consistent with a squamous cell carcinoma or sarcoma	Severe bone destruction	Higher utilization of contrast agent	12 months
Danno et al. [20]	43	Male	NR	Soft palate	NR	NR	NR	NR
Li et al. [21]	48	Male	3 months	Hard palate and soft palate	NR	NR	No higher utilization of contrast agent.	5 months
Saravanam et al. [22]	10	Female	NR	Hard palate, soft palate, uvula, upper part of anterior and posterior tonsillar pillar	T2: mild heterogeneous enhancement	Subtle cortical erosions	NR	3 months
Li et al. [23]	63	Female	4 months	Soft palate	NR	NR	High retention of contrast agent	6 months
Okubo et al. [24]	63	Female	3 months	Mouth floor	NR	NR	NR	NR
Aldoori M, et al. [25]	46	Male	40 days	Tongue	NR	NR	NR	9 months
Our patient	53	Male	2 years	Soft palate, hard palate and bilateral tonsils	T1: hypo-intense signal T2: hyper-intense signal	Subtle cortical erosion	NR	12 months

NR, not reported.

**Table 2:** Characteristic symptoms of KD patients with oral lesions.

	Number of Patients (%)
Total	18 (100.0)
Tumor size	
>3 cm	11 (61.1)
<3 cm	4 (22.2)
NR	3
Oral symptom	
Pigmentation	5 (27.7)
Ulcer	2 (11.1)
Pruritus	1 (5.6)
Eosinophil	
Increased	11 (61.1; ranged 370 to >5000 IU/mL)
Normal	2 (11.1)
NR	5
IgE	
Increased	7 (38.9; ranged 900 to 3150 /IL)
Normal	2 (11.1)
NR	9
Symptoms beyond the head and neck regions	
Subcutaneous tumor	4 (22.2)
Renal disorder	3 (16.7)

NR, not reported.

**Table 3:** Treatment and prognosis of KD patients with oral lesions.

	Number of Patients	Number of Patients with Recurrence (%)
Total	18	2 (11.1)
Initial treatment		
Total resection	4	0
Limited surgery	2	1
Medication	5	1
Combination of surgery and steroid	4	0
Radiotherapy	1 (our patient)	0
NR	2	NR

NR, not reported.

## Data Availability

Not applicable.
